# Implementing and sustaining a mobile medical clinic for prenatal care and sexually transmitted infection prevention in rural Mysore, India

**DOI:** 10.1186/s12879-017-2282-3

**Published:** 2017-03-06

**Authors:** Noah Kojima, Karl Krupp, Kavitha Ravi, Savitha Gowda, Poornima Jaykrishna, Caitlyn Leonardson-Placek, Anand Siddhaiah, Claire C. Bristow, Anjali Arun, Jeffrey D. Klausner, Purnima Madhivanan

**Affiliations:** 1 David Geffen School of Medicine, University of California, Los Angeles, 10833 Le Conte Ave, Los Angeles, 90095 CA USA; 2 Public Health Research Institute of India, 89/B, Ambika, 2nd Cross, 2nd Main, Yadavagiri, Mysuru, Karnataka 570020 India; 3 Robert Stempel College of Public Health and Social Work, Florida International University, 11200 SW 8th Street, Miami, 33199 FL USA; 4 Division of Global Public Health, Department of Medicine, University of California San Diego, 9500 Gilman Dr, La Jolla, 92093 CA USA

**Keywords:** Mobile Health, India, Pregnant, Women, HIV, PMTCT

## Abstract

**Background:**

In rural India, mobile medical clinics are useful models for delivering health promotion, education, and care. Mobile medical clinics use fewer providers for larger catchment areas compared to traditional clinic models in resource limited settings, which is especially useful in areas with shortages of healthcare providers and a wide geographical distribution of patients.

**Methods:**

From 2008 to 2011, we built infrastructure to implement a mobile clinic system to educate rural communities about maternal child health, train community health workers in common safe birthing procedures, and provide comprehensive antenatal care, prevention of mother-to-child transmission (PMTCT) of human immunodeficiency virus (HIV), and testing for specific infections in a large rural catchment area of pregnant women in rural Mysore. This was done using two mobile clinics and one walk-in clinic. Women were tested for HIV, hepatitis B, syphilis, and bacterial vaginosis along with random blood sugar, urine albumin, and anemia. Sociodemographic information, medical, and obstetric history were collected using interviewer-administered questionnaires in the local language, *Kannada*. Data were entered in Microsoft Excel and analyzed using Stata SE 14.1.

**Results:**

During the program period, nearly 700 community workers and 100 health care providers were trained; educational sessions were delivered to over 15,000 men and women and integrated antenatal care and HIV/sexually transmitted infection testing was offered to 3545 pregnant women. There were 22 (0.6%) cases of HIV, 19 (0.5%) cases of hepatitis B, 2 (0.1%) cases of syphilis, and 250 (7.1%) cases of BV, which were identified and treated. Additionally, 1755 (49.5%) cases of moderate to severe anemia and 154 (4.3%) cases of hypertension were identified and treated among the pregnant women tested.

**Conclusions:**

Patient-centered mobile medical clinics are feasible, successful, and acceptable models that can be used to provide quality healthcare to pregnant women in rural and hard-to-reach settings. The high numbers of pregnant women attending mobile medical clinics show that integrated antenatal care with PMTCT services were acceptable and utilized. The program also developed and trained health professionals who continue to remain in those communities.

## Background

India, the world’s largest democracy and second largest country [1], is quickly becoming a global power and is projected to become the third largest global domestic product earner by 2030 according to a 2015 report released by the United States Department of Agriculture Economic Research Service [2]. While India has made great strides to provide healthcare to its over 1.25 billion people [3, 4], it still faces challenges in terms of infectious diseases, including human immunodeficiency virus (HIV), syphilis, and hepatitis B [5, 6], chronic diseases [7], nutritional deficiencies [8], newborn health outcomes [8], health inequality [9, 10], gender inequality, and violence against women [11]. Due to acute healthcare provider shortages, especially in rural areas [12, 13], new strategies are needed to address those underserved communities and populations [14].

India continues to have challenges caring for the needs of underserved women. A report from the World Economic Forum reported that India holds the lowest rank in gender parity of the BRIC (Brazil, Russia, India, and China [countries of newly advanced economic development]) countries (114 of 142 total ranked countries), a ratio of female-to-male labor force participation of 0.36 (134 of 142), showing unequal economic opportunity and participation, a female-to-male literacy rate of 0.68 (126 of 142), and the rank of second to last in terms of health and survival of women (141 of 142) [15]. All of those statistics highlight the state of underserved women in India, who not only need healthcare, but also education and health literacy. Improved access and provision of healthcare is especially important for pregnant women and infants in India.

Given healthcare provider shortages and a wide geographical distribution of rural patients in India, mobile clinics are useful models to provide health services, health promotion, and education to large catchment areas [12, 13, 16, 17]. Our aim was to demonstrate that mobile clinics catering to pregnant women are feasible and acceptable models in rural India, to deliver education and antenatal care with HIV/sexually transmitted infection (STI) testing and management.

## Methods

### Program design and participants

The mobile clinic program started in 2008 in Mysore District, India by the Public Health Research Institute of India (PHRII), a non-profit organization. PHRII is (i) a Public Chartable Trust accredited as a Scientific and Industrial Research Organization by the Ministry of Science and Technology of the Government of India, (ii) has clinical and basic science laboratories capable of processing all required investigations, (iii) operates the Prerana Women’s Clinic, which offers additional services in family planning, reproductive health care, and HIV prevention services to rural, underserved women, (iv) has an ethics review committee, and (v) partners with several community-based organizations including the District Health Office, the District AIDS Prevention and Control program, the National Rural Health Mission, and the Karnataka Health Authority, a division of the Ministry of Health and Family Welfare under the aegis of Government of India. PHRII is located in the south Indian state of Karnataka, which had a high prevalence of HIV [18, 19] and diverse populations living in rural and tribal communities [20].

The population of the state of Karnataka is over 61 million people with 61% living in rural villages [21]. The project targeted rural pregnant women living ten kilometers outside of Mysore city, who consented to health interviews and antenatal care and testing for infections that are transmissible from mother-to-child including HIV.

## *Kisalaya* project (2008–2011)

### Building infrastructure and program design

Traditional birth attendants (TBAs) also called ‘*Dais*’ play an important role in reaching pregnant women who do not have antenatal care, according to the health ministry guidelines [22]. At the time of the project’s inception, it was estimated that 62% of all deliveries in the Mysore district were home-based ones [23], with many TBAs operating without formal training. The *Kisalaya* project aimed to map and train TBAs in Mysore sub-district to reduce perinatal morbidity and mortality associated with home deliveries, increase the number of women receiving counseling and testing for HIV, provide community medical education, and improve prevention of mother-to-child transmission (PMTCT) of HIV.

To develop that infrastructure, one full-time female physician was recruited to manage the program and trained on issues related to PMTCT of HIV and HIV management. In addition women were hire﻿d for positions, which include﻿d another part-time physician,a ﻿staff manager, a data manager, counselors, and community health workers. Counselors and community health workers trained to build rapport with eligible communities. A seven-day training course on PMTCT of HIV was held for all staff members before initiation of the project. A community advisory board, which continues to meet on a regular basis at PHRII, was used to solicit community input.

All villages in the Mysore sub-district were mapped, which included meeting with local village leaders, stakeholders, and TBAs. The geographical coordinates of the residences of TBA were recorded with hand-held GPS devices, as many homes did not have proper residential addresses. The TBAs, if interested, were then invited to participate in an educational session. The three-day seminar session included information on recognition of key health indicators during pregnancy and delivery, understanding HIV and PMTCT of HIV, and referring patients to higher levels of care for obstetric complications in their catchment areas. Delivery kits were provided to further standardize safe-delivery environments with each kit containing plastic sheets for deliveries, gloves, scissors, thread, blades, soap, and an apron.

### Mobile clinics

After building necessary infrastructure, medical services were expanded to provide mobile health consultations for pregnant women. Program staff first met with all community leaders and got their buy-in before initiating activities in any community. Once leaders provided necessary permission and support, an education and awareness program was held and was open to all residents in that community. The time and location for the medical clinic for all pregnant women was announced during the education program and conducted the following day to ensure the families had time to discuss and decide whether or not they would participate. Mobile medical clinics consisted of two vehicles that carried staff and equipment necessary to conduct health clinics and education programs. Those clinics provided education about general reproductive health, prenatal health screenings, nutritional counseling, HIV testing, treatment for reproductive tract infections, and antiretroviral drugs for pregnant women infected with HIV for PMTCT of HIV. Sociodemographic, medical, and obstetric history were collected using an interviewer-administered survey in the local language of *Kannada*, and all women provided urine, blood, and vaginal samples for laboratory assessment of infections (Table 1). Pregnant women were examined by a physician and nurse phlebotomist team before they received prenatal nutritional supplements. All laboratory assessments were conducted at the PHRII laboratory in Mysore city within 24 h of collection. Program counselors reported back the test results with post-test counseling, and referrals within 48 h of the initial encounter. Postnatal and one year follow-up visits were conducted for all women who attended the antenatal clinics.

**Table 1:** Data collected from the *Kisalaya* and Saving Children, Improving Lives mobile clinic projects, Mysore, India, 2008-2014

Surveys collected
Social
Sociodemographics
Partner characteristics
Work and economic status
Sexual history
Knowledge of HIV/AIDS
History of domestic violence
History of sexually transmitted infections
Clinical
Past medical history
Obstetric history
General health
Physical exam
Factors associated with institutional delivery
History of antenatal depression
Clinical samples
Vitals
Blood was tested for:
Blood type
Random blood sugar
HIV
Hepatitis B
Syphilis
Anemia
Malaria^a^
Urine was tested for:
Blood sugar
Albumin
Vaginal samples were tested for:
Bacterial vaginosis
Stool samples^a^ were tested for:
Helminthic infections
Interviews and samples collected during the mobile clinic projects in Mysore district, India
^a^only tested in a small random sample of the SCIL cohort

## Saving Children, Improving Lives (SCIL) project (2011–2014)

### Program design and updated methods

The SCIL project focused on continuing education and mobile health clinics for rural pregnant women, while updating protocols by applying lessons learned from the *Kisalaya* project. The project had an additional social mobilization component that strengthened community networks for pregnant women [24]. The SCIL program utilized local peer groups to encourage women to visit the mobile medical clinics and assist with follow-up postnatal visits. The community-member support through self-help groups was successful in motivating pregnant women to seek health care services ([24]: A paper describing methodology of social mobilization, but does not provide program results).

### Community education

Varied approaches were used to engage and educate the community depending on their needs. Strategies included street theater, interactive presentations, and videos shown during the town hall meetings, which normally lasted two hours, on the day prior to mobile medical clinics. Information on the importance of birthing preparedness, antenatal care, HIV testing, institutional deliveries, and recognizing key health indicators during pregnancy was provided [24]. Those programs were open to all members of the community including extended family members of the pregnant woman. Efforts were made to involve key community stakeholders and gain their support for the program.

### Data collection

Data were collected from all pregnant women attending the medical clinics by PHRII program staff (Table 1). Sociodemographic, medical, and obstetric history, knowledge, attitude and beliefs about HIV, and factors influencing institutional delivery were collected using paper questionnaires in an interviewer-administered survey in *Kannada*. Urine, blood, vaginal, and stool samples were collected as a part of antenatal care for screening for infections. Follow-up visits were conducted within 15 days of birth, at six months, and one year after delivery. All infections that were diagnosed were either treated immediately or referred to tertiary care centers for further management. Data were entered onto a computer database by data entry staff.

All laboratory testing was conducted at the PHRII laboratory shortly after collection. Sera samples were tested for HIV (ErbaLisa HIV 1 + 2 Gen3 [Erba Mannheim, Mannheim, Germany] and HIV TRI-DOT [J. Mitra & Co Pvt. Ltd., New Delhi, India]), hepatitis B (ErbaLisa Hepatitis B surface antibody test [Erba Mannheim, Mannheim, Germany]), and syphilis (Rapid Plasma Reagin (RPR) [Span Diagnostics, Surat, India]).

All data were entered into Microsoft Access (Redmond, WA) and analyzed using StataSE 14.1 (College Station, TX).

## Results

### Kisalaya project

From 2008 to 2011, the *Kisalaya* project identified, mapped, and interviewed 417 TBAs. Collaborating with the National Rural Health Mission, PHRII staff trained 77 ANMs and 126 Accredited Social Health Advocates, whose total catchment area was over 250,000 persons in rural Mysore (Table 2). Additionally, 423 Community Health Workers were trained in safe birthing practices and PMTCT of HIV. The program trained and funded 45 PHRII affiliated staff. PHRII staff conducted 141 education and awareness programs, attended by 5161 community members, of which 4212 were women and 899 were men.

**Table 2:** Training outcomes from the *Kisalaya* and Saving Children, Improving Lives mobile medical clinic projects, Mysore, India, 2008-2014

	Kisalaya project (2008–2011)	Saving Children, Improving Lives project (2011–2014)
	N (%)	N (%)
Public Health Research Institute of India staff		
Physicians	4 (9%)	4 (8%)
Nurses	3 (7%)	3 (6%)
Counselors	7 (16%)	8 (16%)
Drivers	3 (7%)	3 (6%)
Outreach workers	5 (11%)	10 (20%)
Laboratory staff	5 (11%)	12 (24%)
Core staff	6 (13%)	6 (12%)
Visiting scholars	12 (27%)	5 (10%)
Total	45	51
Educational programs held	141	244
Women	4262 (83%)	6257 (62%)
Men	899 (17%)	3768 (38%)
Total	5161	10,025
Community workers		
Community health workers	423 (64%)	-
Accredited social health advocates	126 (19%)	60 (32%)
Auxiliary nurse midwives	77 (12%)	-
Traditional birth attendants	40 (6%)	-
Microeconomic self-help groups	-	129 (68%)
Total	666	189

With 92 mobile medical clinic consultations conducted during that period, a total of 1675 (76%) from the 2211 pregnancies in the 144 villages received antenatal care from *Kisalaya* program with 1639 (97.85%) women consenting to counseling and testing for HIV along with all the other routine investigations (Table 3). All women who were tested for HIV also received their post-test counseling and test results (100%). Of the tested women, 14 (0.9%) were found to have new HIV infections and received management to prevent vertical transmission of HIV. Those women were also accompanied to the tertiary care facility to ensure that they had access to antiretroviral therapy through the government sponsored treatment programs. Of the newborns born to HIV-positive mothers, 7 (50%) were HIV negative, 3 (21%) were deceased, and 4 (29%) did not have data available. Six women (0.4%) tested positive for hepatitis B and two (0.1%) women tested positive for syphilis serology. Bacterial vaginosis was diagnosed in 97 (5.9%) women. Among the women, 306 (18.7%) were mildly anemic, 381 (23.2%) had moderate to severe anemia, and 94 (5.7%) had hypertension.

**Table 3:** Medical outcomes from the *Kisalaya* and Saving Children, Improving Lives mobile medical clinic projects, Mysore, India, 2008-2014

	*Kisalaya p*roject (2008–2011)	Saving Children, Improving Lives project (2011–2014)
	N (%)	N (%)
Mobile medical clinics held	92	223
Women seen	1675	1948
Women tested	1639	1906
1st follow-up done after delivery	1675 (100%)	1944 (99.7%)
6 month follow-up	-	1870 (96%)
12 month follow-up	1675 (100%)	1780 (91%)
Reactive HIV serology	14 (0.9%)	8 (0.4%)
Reactive Hepatitis-B serology	6 (0.4%)	13 (0.7%)
Reactive Syphilis serology	2 (0.1%)	0 (0.0%)
Anemic	687 (41.9%)	1068 (56.0%)
Mild Anemia	306 (18.7%)	642 (33.7%)
Moderate to Severe Anemia	381 (23.2%)	426 (22.4%)
Pre-eclampsia	94 (5.7%)	60 (3.1%)
Bacterial Vaginosis	97 (5.9%)	153 (8.0%)

The *Kisalaya* project had two follow-ups (Table 3). Of the 1675 pregnant women initially seen, all (100%) of the mothe﻿r-infant dyads attended both follow-up visits; the first follow-up occurred within 45 days of birth and the second around one year after birth. Of the pregnancy outcomes, 97 (5.8%) reported a miscarriage and another 13 (0.8%) had stillbirths. Of the newborns, 1576 (94%) were delivered at a health facility, 123 (7.3%) were admitted to a neonatal intensive care unit, 74 (4.4%) suffered from complications, and 45 (2.7%) were subsequently hospitalized.

### SCIL project

From 2011 to 2014, the SCIL mobile clinic project saw 1948 pregnant women and conducted 244 education and awareness programs for 10,025 community members (Table 2). The program trained and supported 51 PHRII affiliated staff. HIV testing was offered to 1906 (97.8%) women (Table 3) and all women (100%) received post-test counseling and test results. Eight women tested positive for HIV (0.4%) and received medication for PMTCT of HIV. Of the newborns born to HIV-positive mothers, 2 (25%) were HIV negative, 2 (25%) were deceased, and 4 (50%) did not have data available. Among the women, 13 (0.7%) had positive hepatitis B serology testing, none had positive syphilis serology﻿ testing﻿, and 153 (8.0%) had bacterial vaginosis. Additionally, 642 (33.7%) women had mild anemia, 426 (22.4%) women had moderate to severe anemia, and 60 (3.1%) women had hypertension.

The SCIL project had three follow-ups (Table 3). Of the 1948 pregnant women seen, 99.7% of the mother-infant dyads were seen within 45 days of birth, 96% were seen at follow-up 6 months after birth, and 91% at follow-up 1 year after birth. Among the pregnant women, 152 (7.8%) had miscarriages and 16 (0.8%) had stillbirths. Of the newborns, 1692 (87%) were delivered at a health facility, 155 (8.0%) were admitted to a neonatal intensive care unit, 58 (3.0%) suffered from complications, and 22 (1.1%) were subsequently hospitalized.

## Discussion

The *Kisalaya* and SCIL projects demonstrated that mobile clinics for pregnant women are feasible and acceptable models to deliver education about safe child delivery and antenatal care with HIV/STI testing in rural India. The high numbers of pregnant women attending the medical clinics show that integrated antenatal care with HIV testing services were utilized by rural pregnant women, if those services were made accessible in their communities. The high follow-up rates for the *Kisalaya* and SCIL projects show that it is possible to follow mother-infant dyads when providing health services in rural settings using mobile medical clinics.

Additionally, the projects developed and trained many health professionals. By hiring women, some of whom were married or pregnant, PHRII provided opportunities that broke traditional gender roles, allowing women to gain upward job-market mobility, learning about their own health, and sharing what they had learnt with their communities.

Opportunities for women are necessary in a country where gender inequality and discrimination are still ever-present in all levels of society [25]. Additionally due to reports of boundary violations in provider-patient relationships in India [26], health care that is provided by women for women could be more patient-centered. Research has shown that female patients have a preference for female health providers [27].

A challenge of implementing mobile health clinics was community participation and engagement. A prior study conducted among *Kisalaya* program participants found that reasons for breaking appointments included: (a) pregnant women leaving the village to stay at their maternal home for the rest of their pregnancy; (b) prior delivery; (c) not being in the village at the time of the medical clinic; (d) having an abortion/miscarriage; (e) receiving antenatal care or other medical service at another hospital at the time of medical clinic consultation; (f) relocating to another village; (g) refusing services out of fear; (h) caste stigma; and (i) not being confirmed to be pregnant [24]. It was not possible for our group to be able to continue to follow those women with our limited resources.

There were operational challenges in the implementation and continuation of the programs. Community involvement initially was a challenge, but this changed in the SCIL project. In the SCIL project, a social mobilization component with local peer groups was added to encourage and provide support to pregnant women to visit the mobile medical clinics and assist with follow-up postnatal visits. The program was structured to employ one allopathic doctor and one AYUSH, Ayurveda, Yoga, Unani, Siddha, and Homeopathy, doctor experienced in maternal child health services. Over the duration of the program, there was higher physician turnover than nurse turnover, leading to additional physician training, reflected in Table 2. Other reasons for staff turnover include: trained and experienced staff leaving for higher paying jobs and women being asked by their husbands to stop working. Biomedical waste from program activit﻿ies was brought back to PHRII and then disposed on a bi-weekly basis. Programmatic funds were used for maintenance of automobiles and laboratory and field equipment.

Our objective was to examine the feasibility and acceptability of carrying out a program in rural communities, where access to healthcare services is limited. Further research is needed to evaluate the effectiveness of the intervention with an experimental study design. Continuation of those mobile clinic services is needed to improve birth outcomes, reduce the spread of infectious diseases, and increase health education among those populations.

## Conclusions

India, the world’s largest democracy and second largest country, needs to continue investing in its health care infrastructure for its over 1.25 billion people. Healthcare in India still faces challenges, but solutions like mobile clinic models could be used to continue to improve the lives of pregnant women and children in hard to reach communities. We have shown mobile clinics are a feasible and acceptable model to deliver healthcare in rural India to provide education, antenatal care, and management of vertically transmitted infections. After completion of *Kisalaya* and SCIL projects, PHRII operated mobile clinics continue to provide services to resource limited communities, while empowering women, as patients and professionals.

## Abbreviations

ANMs: Auxiliary nurse midwives
PHRII: Public Health Research Institute of India
PMTCT: Prevention of mother-to-child transmission
TBAs: Traditional birth attendants
